# Aging Markers in Equine Red Blood Cells

**DOI:** 10.3389/fphys.2019.00893

**Published:** 2019-07-17

**Authors:** Sandra Kämpf, Elena Seiler, Jolanta Bujok, Regina Hofmann-Lehmann, Barbara Riond, Asya Makhro, Anna Bogdanova

**Affiliations:** ^1^Red Blood Cell Research Group, Vetsuisse Faculty, Institute of Veterinary Physiology, University of Zurich, Zürich, Switzerland; ^2^Vetsuisse Faculty, University of Bern, Bern, Switzerland; ^3^Institute of Animal Physiology, Wrocław University of Environmental and Life Sciences, Wrocław, Poland; ^4^Clinical Laboratory, Vetsuisse Faculty, University of Zurich, Zürich, Switzerland; ^5^The Zurich Center for Integrative Human Physiology (ZIHP), Zürich, Switzerland

**Keywords:** horse red blood cells, aging, senescence, calcium, membrane loss

## Abstract

Detection of hematopoietic activity in horses is a challenge due to the lack of cells carrying reticulocyte markers such as RNA remnants or CD71 in the circulation. In this study, we fractionated equine red cells according to their density and analyzed the cells forming low (L), medium (M), and high (H) density fractions for markers of aging such as membrane loss, oxidation, and alterations in the intracellular free Ca^2+^ levels. Cells forming L and M fraction were highly heterogeneous in projected areas and shapes, and had higher propensity to swell in response to hypo-osmotic challenge than the cells from the H fraction. The densest cells were deprived of band 3 protein compared to the cells within L or M fraction. Furthermore, the equine red cells from the H fraction were hyper-oxidized compared to the cells within M and L fractions as follows from an increase in autofluorescence characteristic for oxidized damaged hemoglobin and from thiol oxidation as detected using monobromobimane. The lightest cells showed lower free thiol content compared to the red blood cells from the M fraction, but did not contain oxidized hemoglobin. Finally, the majority of red blood cells forming L, M, and H fraction prominently differed from each other in intracellular free Ca^2+^ levels and its distribution within the cells. Based on the obtained findings, we suggest that intraerythrocytic Ca^2+^ levels and its subcellular distribution, eosin-5-maleimide binding test for band 3 abundance, and autofluorescence of cells along with the changes in red blood cell indices, distribution width and creatine levels may become potential markers of regenerative erythropoiesis in horses. Validation of the power of these potential markers of red cell aging is pending.

## Introduction

Equine red blood cells (RBCs) are surviving in the circulation for as long as 140–150 days (Carter et al., 1974) being exposed to shear stress, oxidation, and hyperthermia associated with high physical activity of these animals. Multiple attempts to detect reticulocytes (RNA-positive cells or cells carrying transferrin receptor) in peripheral blood of horses failed even in the studies, where stress erythropoiesis was induced by phlebotomy, administration of phenylhydrazine, or erythropoietin administration (Lumsden et al., 1975a,b; Shull, 1981; Radin et al., 1986; Cooper et al., 2005). Two possible reasons for that include (1) maturation of reticulocytes in bone marrow, and (2) facilitated clearance of surface markers such as transferrin receptor from the circulating young cells. Indeed, although transferrin receptor was not detected on the surface of circulating cells, it was recently found in the exosomes (vesicles) isolated from equine blood plasma (Rout et al., 2015). Stimulation of *de novo* RBC production in horses was associated with an increase in heterogeneity reflected in an increase in red cell distribution width (RDW), and in most cases, with an increase in mean corpuscular volume (MCV) (Lumsden et al., 1975a,b; Radin et al., 1986; Cooper et al., 2005; Singh et al., 2007; McKeever et al., 2016). Detection of RBC life-span in horses in which stress erythropoiesis was induced by phlebotomy or erythropoietin administration, revealed a trend to faster clearance of cells produced upon stimulation of their *de novo* production (Lumsden et al., 1975a,b).

The changes in RDW and MCV alone are insufficient as self-standing markers of upregulated hematopoietic activity as these parameters are variable among different breeds of horses, their age, and physical activity. Moreover, they show inter-laboratory variability and depend on the hematological analyzer used (Lording, 2008; Bauer et al., 2012; Satue et al., 2012). Regenerative erythropoiesis is only diagnosed when proven by the examination of bone marrow aspirates (Schalm, 1975, 1981; Lording, 2008; Grondin and Dewitt, 2010). However, contamination of bone marrow aspirates with peripheral blood makes such diagnosis prone to artefactual readouts (Schalm, 1975).

Based on our extensive knowledge of human RBC aging, we have undertaken an attempt to identify a set of possible markers of RBC aging (density, redox state, membrane loss, morphometry, Ca^2+^ levels, and compartmentalization). Along with the changes in RBC indices, this set of markers would be robust and reliable to complement or replace bone marrow aspirate cytology. Aging of healthy human RBC is associated with a gradual membrane loss and increase in RBC density. The existence of RBC fractions of low (L), medium (M), and high (H) density in horse blood was earlier on reported by Wu et al. (1983), and related to the creatine concentration, and, therefore, to the RBC age. We have adapted the measurements of such age-related parameters as intracellular Ca^2+^, band 3 protein abundance, and responsiveness to osmotic challenge, as well as intracellular reduced thiol content (Piccinini et al., 1995; Bogdanova et al., 2013; Lutz and Bogdanova, 2013; Ciana et al., 2017a,b) for detection by means of flow cytometry. This technique is often applied for detection of clinically relevant parameters in hematological laboratories.

## Materials and Methods

Equine heparinized blood samples from 19 horses were obtained from Clinical Laboratory of the Vetsuisse Faculty, University of Zurich. The samples were collected by veterinary practitioners as a part of diagnostic workup and sent to the laboratory for routine diagnostic purposes. Leftovers of the samples were used, and no additional blood volume was collected for the current study. No ethical approval was necessary for this study in compliance with the Swiss regulations. Blood samples were processed for analysis, less than 12 h after blood withdrawal. Human blood samples were collected from ten young male adults (elder than 18 years old) within the study on neocytolysis (DFG-SNF, # 320030E_180227). All participants gave their written informed consent prior to the study onset. The study protocol was approved by the Ethics committee of the Medical Department of the University of Heidelberg (S-066/2018). Blood was collected by the authorized medical practitioner at the Medical Department and immediately transported by couriers to the processing site at the University of Zurich at constant temperature no longer than 5 h, where it was immediately processed. Blood was fractionated on Percoll gradient and Ca^2+^ levels were detected in fractions using flow cytometry and fluorescence microscopy as stated elsewhere (Makhro et al., 2017).

RBCs were pelleted and washed three times with the plasma-like medium of the following composition (in mM) 140 NaCl, 4 KCl, 0.75 MgSO_4_, 10 glucose, 0.015 ZnCl_2_, 0.2 glycine, 0.2 Na-glutamate, 0.1 arginine, 0.6 glutamine, 0.2 alanine, 20 HEPES – imidazole (pH 7.4 at room temperature), and 0.1% bovine serum albumin. This medium was used throughout this study along with the phosphate buffer (PBS).

### Separation of Equine Red Blood Cells According to Their Density Using Centrifugation in Percoll Density Gradient

Percoll (GE Healthcare Life Sciences) was mixed with 10X PBS (1.37 M NaCl, 27 mM KCl, 100 mM Na_2_HPO_4_, 17.6 mM KH_2_PO_4_, pH 7.2–7.4 at room temperature) in proportion 9:1 to obtain isotonic Percoll solution. RBC suspension in 1X PBS was carefully layered on top of 13 ml of Percoll solution and spun using Sorwall Lynx 4,000 Centrifuge (Thermo Fisher Scientific) equipped with A22-24x16 rotor at 10,000 ×*g* for 40 min at 30–33°C. Distribution of RBCs into low (L), medium (M), and high (H) density fractions was recorded photographically and cells forming these fractions were then harvested and washed three times from Percoll using the plasma-like medium. Thereafter, RBCs were re-suspended in the same medium to a 40–50% Hct were prepared on and used for further studies (flow cytometry, microscopy, or RBC membrane isolation).

### Morphological Characterization and Ca^2+^ levels in Red Blood Cells Forming L, M, and H Fractions Using Fluorescence Microscopy

An aliquot of RBC suspension (1 μl) was added to 1 ml of plasma-like medium supplemented with 2 mM CaCl_2_ and 2 μM fluo-4AM (Thermo Fischer Scientific) and incubated for 1 h in the darkness. Thereafter, the samples were transferred into the imaging chambers and the bright field images were taken with the Axiovert 200M fluorescent microscope (Carl Zeiss Jena GmbH, Jena, Germany) equipped with x100 oil objective along with images of Fluo-4 fluorescence. All measurements were performed in triplicates, for each sample, 10 fields were imaged. Images were analyzed using CellFinder software [copyright of Maxim Makhinya (Makhro et al., 2017)].

In a separate set of experiments, RBCs loaded with Fluo-4 were re-suspended in Ca^2+^-free medium and Ca^2+^ uptake following administration of CaCl_2_ (from 1 M stock solution to reach the final concentration of 1.8 mM) was monitored over 12 min. Bright field and fluorescence images were taken for the same field every minute and kinetics of morphological alterations and Ca^2+^ levels in RBCs responding to Ca^2+^ administration was analyzed using the CellFinder software (for details, see Makhro et al., 2017).

### Flow Cytometry

RBCs suspension of ~40–50% hematocrit was prepared in plasma-like medium and 2 μl aliquot of it was mixed with 1 ml of the plasma-like medium containing the following fluorophores: acridine orange (BD Retic-Count™), annexin conjugated with eFluo-450 (eBioscience/Affymetrix at Thermo Fisher Scientific), fluo-4 AM (2 μM), and monobromobimane (10 μM). Separate samples without staining were used to detect autofluorescence of RBCs in all channels (excitation at 488, 635, and 405 nm), which was used as a background reference as well as for estimation of hemoglobin oxidation. The cells were incubated with fluorophores for 1 h in the darkness.

In addition, 5 μl of RBC suspension were incubated in 50 μl of CaCl_2_-containing plasma-like medium containing 0.5 mg/ml eosin 5-maleimide (EMA, Merck KGaA, Darmstadt, Germany) for 1 h in the darkness. The excess of EMA was then washed away during triple washing in plasma-like medium (30 s, 3,000 ×*g*), and the cells were finally re-suspended in 1 ml of the same medium.

Fluorescence from the fluorochromes was detected using Gallios flow cytometer (Becton Coulter, Indianapolis, IN, USA) equipped with 525/30 BP, 669/20 BP, and 450/50 BP filters. Recordings from 100,000 cells per sample were analyzed using Kaluza analysis software (Beckman Coulter Life Sciences, Indianapolis, IN, USA).

Mechano-sensitive Ca^2+^ uptake by equine RBCs was confirmed by us in a preliminary set of experiments and monitored in fractions as a time course over 2–5 min by flow cytometry. RBCs pre-loaded with fluo-4 were stimulated to swell by mixing of cell suspension in isotonic buffer with distilled water (2:1). Response to acute decrease in osmolarity from 330 to 220 mOsm was recorded as a change in side scatter and the alteration in Ca^2+^-dependent fluorescence of Fluo-4 fluorochrome.

### Band 4.1a:b Ratio

Membranes were isolated from the cells forming L, M, and H fraction and proteins separated on the SDS PAGE gel with the subsequent visualization using Coomassie blue staining. Images of the gels were taken using a CoolSNAP_cf_ camera (Photometrics, Tucson, AZ, USA) equipped with Sigma 50 mm 1:2.8 DC MACROD objective (Hama GmbH & Co KG, Monheim, Germany). Image analysis was performed using MCID image analysis software package for gel densitometry. Identity of equine protein(s) forming a double-band corresponding in electrophoretic mobility to the human band 4.1 was assessed using mass spectrometry.

### Mass Spectrometry

Gel bands corresponding to the band 4.1a and b were carefully harvested, cut into small pieces, and washed two times with 100 μl 100 mM NH_4_HCO_3_/50% acetonitrile, and one time with 50 μl acetonitrile alone. All three supernatants were discarded. The proteins were then digested using 10 μl trypsin (5 ng/μl in a buffer containing 10 mM Tris and 2 mM CaCl_2_, pH 8.2) in 30 μl of the same Tris-CaCl_2_ buffer for 30 min at 60°C in a microwave. Supernatant was then removed, and gel pieces extracted one time with 150 μl 0.1% TFA/50% acetonitrile. All supernatants were combined and dried. Samples were dissolved in 20 μl 0.1% formic acid and transferred to autosampler vials for LC/MS/MS. About 3 μl (samples 1, 3, 7, and 9) or 5 μl (2, 4, 5, 6, 8, and 10) were injected and analyzed. Database searches were performed by using the Mascot (SwissProtr, all species; Trembl, mammalian) and PEAKS (*de novo* sequencing and search against a database containing the sequences of *Equus caballus* extracted from the NCBI database) search programs and search results were summarized in Scaffold matrix.

### Statistics

Statistical module of SigmaPlot v.13 was used for analysis of variance and differences. It included characterization of the normality of distribution (Shapiro-Wilk test) with the following choice of parametric or nonparametric analysis tools. Wilcoxon signed rank test and the Repeated Measures ANOVA on Ranks was used in majority of cases. For more details, see Figure legends.

## Results

### Separation of Horse Red Blood Cells Into Fractions of Low, Medium, and High Density

**Figure 1:**
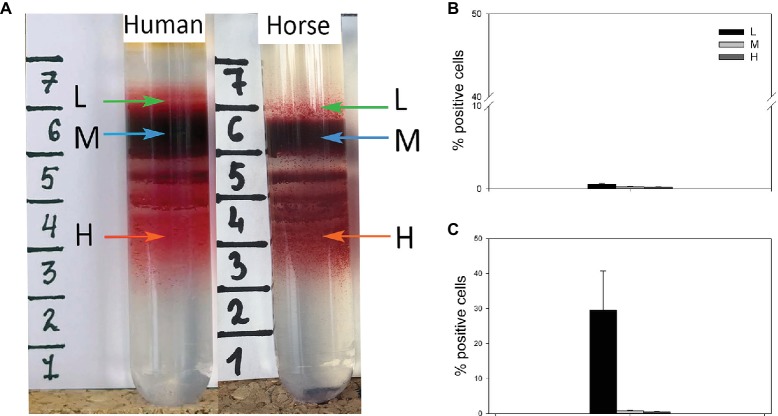
**(A)** Distribution of RBCs of human and horse within Percoll density gradient and the abundance of RNA+ cells within the light (L), medium (M) and high (H) density fractions of horse **(B)** and human **(C)** blood. Data are means ± SD for four humans and four horses.

Best results for human RBC separation were obtained using PBS-based isosmotic Percoll solution with the density of 1.1126 kg/L (Figure 1A). Optimal conditions for successful separation of horse equine RBC into L, M, and H fractions (Figure 1A) were achieved on the self-forming PBS-based isosmotic Percoll density gradient of higher density of 1.124 kg/L. The differences in density between the species reflected the higher mean cells hemoglobin concentration (MCHC) and the smaller cell size as follows from the mean cell volume (MCV) and the RBC diameter of horse cells compared to those of humans (Table 1). Clearly separated, L, M, and H fractions of equine and human RBCs (Figure 1A) were harvested and the cells forming them stained for RNA as a reticulocyte marker. Whereas in equine L fraction of equine RBCs, only 0.54 ± 0.14% were positive for RNA (Figure 1B), human L fraction contained 29.5 ± 11.2% of cells positive for RNA (Figure 1C).

**Table 1:** RBC indices for horses and humans.

Parameter	Horse RBCs (*T* 140–170 days) (Lording, 2008)		Human RBCs (*T* 100–120 days) (Turgeon, 2005)	
	Warm-blooded	Cold-blooded	Female	Male
Ery, 10^12^/L	8.2–12.2	5.5–9.5	4.2–5.4	4.6–6.2
Hb, g/L	130–170	80–140	115–160	140–180
MCHC, g Hb/L	330–390	320–380	300–340	300–340
MCV, fL	36–50	40–48	80–95	80–95
Diameter, μm	5.7		6.2–8.2 (7.2)	
RDW,%	14–25		11.5–14.5	

**Figure 2:**
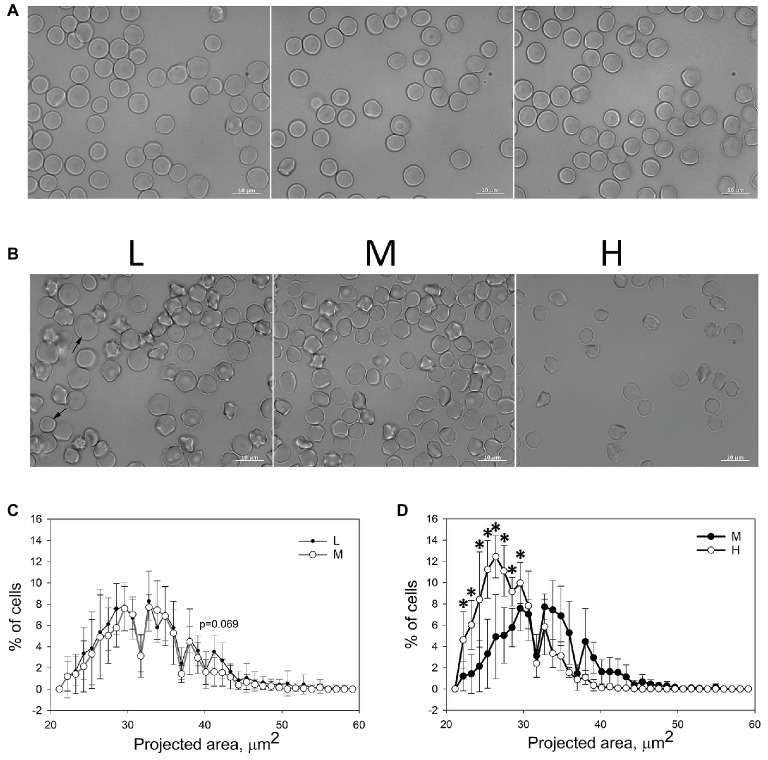
Microscopic evaluation of equine RBC. **(A)** Morphological appearance of equine RBCs in whole blood of three different horses. **(B)** Morphology of equine RBCs forming L, M and H fractions. **(C,D)** Binned projected area assessed for RBCs forming L, M, and H fractions for six horses ±SD. Wilcoxon signed rank test was used for statistical analysis and the two-tailed *p* values are presented as stars (*signifies *p* < 0.05).

Bright-field images of equine RBCs from whole blood (Figure 2A) as well as of cells forming L, M, and H fractions were obtained (Figure 2B). The images were analyzed and the cellular projected areas and anisotropy (ellipticity) were obtained. In agreement with earlier reports (Lording, 2008), equine RBCs had less pronounced central pallor, compared to the human cells (Figure 2A). Mild echinocytosis was seen in all fractions of several, but not all, horses (Figure 2B). In the L and M fractions, RBCs with a broad variety of projected areas (Figures 2C,D) were observed. Within the H fraction only discocytes with small projected areas were observed (Figure 2D). Detailed probability density analysis of the distribution of projected areas revealed that L and M fractions contain the cells of similar sizes and shapes (Figure 2C). A small population of cells within the L fraction with a projected area of 42–45 μm^2^ was exceeding that in the M fraction (*p* = 0.069). The majority of RBCs forming the H fraction had significantly smaller projection areas than the cells forming or M fraction (Figure 2D).

RBC ellipticity (longest to shortest diameter ratio) was gradually increasing from L to H fractions making up (mean ± SD): 1.070 ± 0.018, 1.072 ± 0.028, and 1.078 ± 0.025 for L, M, and H fractions, respectively. However, the significant differences in eccentricity were only recorded between the L and H fractions, showing that the cells with lower density were also more “round” (*p* < 0.05 Mann-Whitney Rank Sum Test).

**Figure 3:**
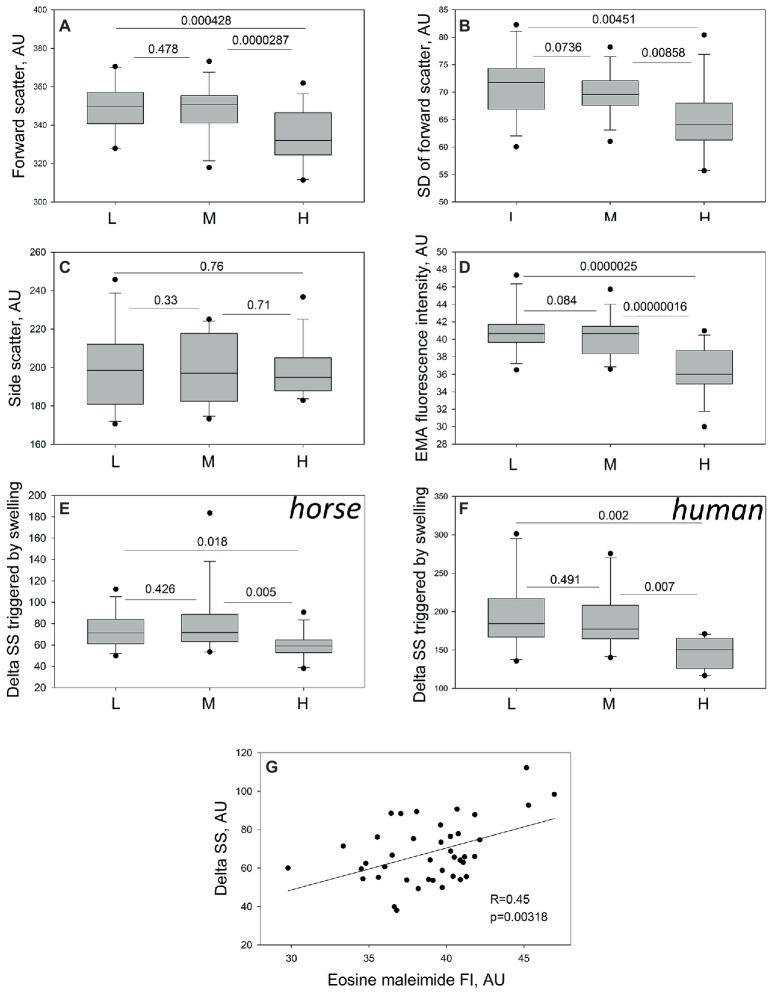
Assessment of the RBC sphericity and membrane loss in equine RBCs by flow cytometry. Forward scatter **(A)** and its variance **(B)**, side scatter **(C)** and band 3 abundance **(D)** in equine RBCs forming L, M, and H fractions of 13 different animals. Changes in side scatter (delta SS) in horse **(E)** and human **(F)** RBCs forming L, M, and H fractions to hypo-osmotic challenge in 13 horses and 10 healthy humans. **(G)** Product Moment Correlation between the EMA staining intensity and delta SS in equine RBCs of 13 horses. For 41 data-points *R* = 0.450, and *p* = 0.00318 were calculated. Wilcoxon signed rank test was used for statistical analysis and the two-tailed “*p*” are presence.

Forward (FS) and side (SS) scatter and their variances (SDs) were used as an indirect indicator of RBC shape heterogeneity and sphericity. The cells forming the H fraction differed from the cells in M and L fraction by having lower FS, whereas side scatter did not differ between the fractions (Figures 3A,C). In line with the data obtained from morphometry analysis (Figure 2D), the densest RBCs appeared more homogeneous than those from the M and L fractions, as followed from reduction in variance of FS (FS SD, Figure 3B). One more special feature of the cells forming the H fraction was a decrease in band 3 abundance compared to the other fractions (Figure 3D).

Functional test for the ability of RBCs to change their shape to more spherical in response to acute hypo-osmotic stress, expressed as a change in SS. As follows from Figure 3E, equine RBCs of the H fraction were less responsive to hypo-osmotic stimulation compared to the cells of L and M fractions. Similar to those of horses, RBCs forming the H fraction in humans were also limited in swelling propensity compared to those from the L and M fractions (Figure 3F). The ability of RBCs to respond to hypoosmotic stress (delta SS) correlated positively (*p* = 0.00318, Pearson Product Moment Correlation) with the band 3 abundance (Figure 3G).

### Intracellular Free Ca^2+^ Content

**Figure 4:**
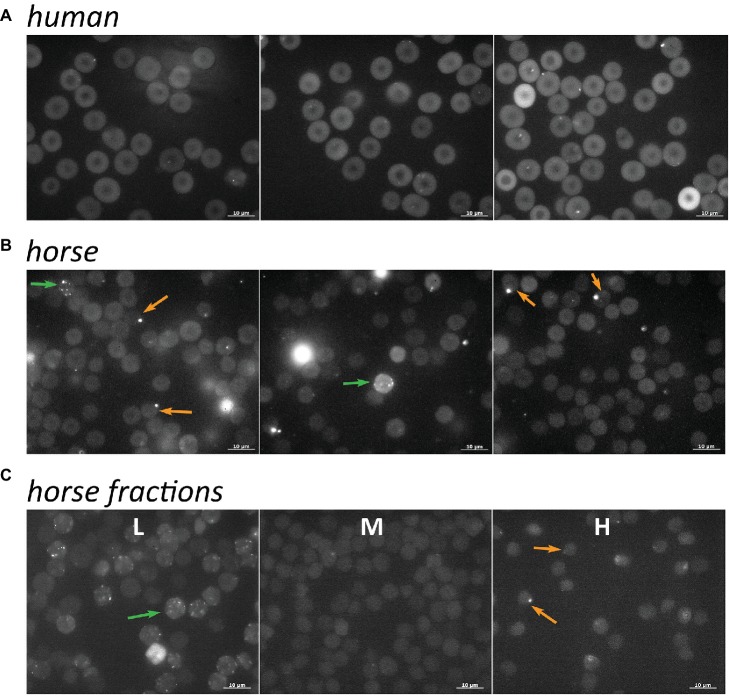
Distribution of intracellular free Ca^2+^ in non-fractionated RBCs of humans (**A**, three different donors) and horses (**B**, three different horses) from whole blood. **(C)** Ca^2+^ distribution in equine RBCs forming L, M, and H fractions. Green arrows indicate RBCs with high basal fluorescence intensity and multiple small Ca^2+^-filled vesicles and orange arrows show the cells with low basal fluorescence and a single large compartment filled with Ca^2+^.

**Figure 5:**
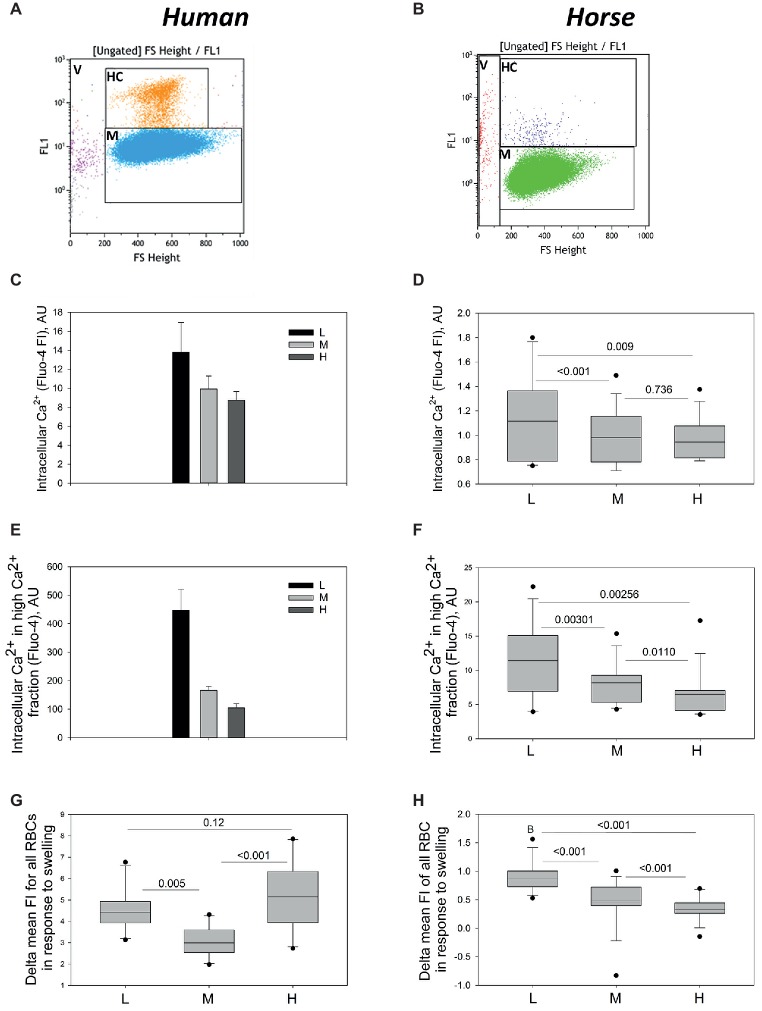
Intracellular Ca^2+^ in L, M and H fraction monitored using flow cytometry. Gating for High Ca^2+^ (HC) and Main (M) population of human **(A)** and equine **(B)** RBCs. **(C)** Fluo-4 fluorescence intensity for the human cells forming L, M, and H fractions (M and H populations). Data are means for four humans ±SD. Fluo-4 fluorescence intensity for the M **(D)** and H **(E)** populations equine RBCs forming L, M, and H fractions (M and H populations). Data are obtained for 13 horses. Increase in fluo-4 fluorescence intensity (delta FI) in equine (panel **F**, *n* = 13) and human (panel **G**, *n* = 10). RBCs from L, M, and H fraction in response to hypoosmotic challenge.

Comparison the fluo-4 readouts obtained by micro fluorescence imaging of unfractionated RBCs (whole blood) of humans (Figure 4A) and horses (Figure 4B) reveals several species-specific features. The fluorescence intensity in human cells is higher than that in RBCs of horses. This observation is at least in part explained by the increased quenching of the signal by hemoglobin in equine cells due to the higher MCHC (Table 1), but may also reflect the higher levels of free Ca^2+^ in human cells (see also Figure 5). Furthermore, higher inter- and intra-cellular heterogeneity in fluo-4 fluorescence was observed in equine RBCs compared to the human ones. In human RBCs, some middle-sized vesicles may be seen in some cells. The abundance of such cells varies between the healthy donors (compare the panels in Figure 4A). In non-fractionated equine RBCs (Figure 4B), three types of cells are always present: those with low basal fluorescence and no vesicles or one single large vesicle (highlighted with orange arrows) and the ones with high intracellular free Ca^2+^ and multiple smaller vesicles (highlighted with green arrows). Fractionation resulted in accumulation of the cells with higher Ca^2+^ and multiple small Ca^2+^-filled vesicles in the L fraction (Figure 4C). Cells with a single larger Ca^2+^-filled compartment were only found in the H fraction (Figure 4C).

To achieve higher throughput in quantification of the whole-cell fluorescence intensity, flow cytometry was used and the readouts for 100,000 cells per sample were maintained. As follows from the representative dot-plots, the absolute levels of fluo-4-derived fluorescence intensity for human cells (Figure 5A) exceeded that for equine RBCs (Figure 5B). Two sub-populations of RBCs were detected is each density fraction in both human and equine samples: main sub-population (gates M in Figures 5A,B) and a smaller sub-population with High Ca^2+^ levels (gates HC in Figures 5A,B). Both human and horse samples also contained Ca^2+^-loaded Vesicles (gates V in Figures 5A,B). The human and equine RBCs forming the L fraction’s M population were presented with the maximal fluorescence intensity of fluo-4 [Figure 5C (human) and Figure 5D (equine)]. The M and H fractions did not differ from each other and were both less fluorescent the lightest cells. Fluorescence intensity of the RBCs forming the H population decreased with an increase in density (Figures 5E,F).

Hypo-osmotic challenge is known to cause activation of mechano-sensitive Ca^2+^ uptake in RBCs of humans (Cahalan et al., 2015; Fermo et al., 2017). Increase in fluo-4 signal triggered by swelling (delta FI) in human RBCs was similar in L and H fractions, and lower in the M fraction (Figure 5G). Delta FI in equine RBCs was smaller in absolute values and maximal in the cells forming L fraction. It decreased progressively with increasing density (Figure 5H).

### Redox State Indicators

**Figure 6:**
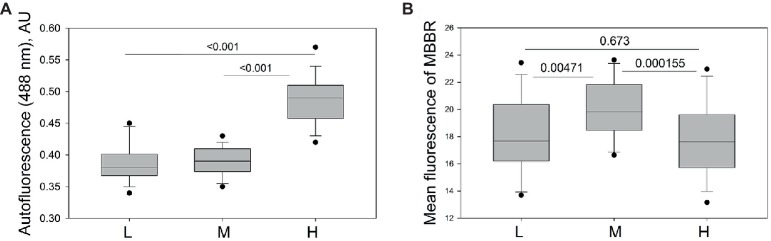
Markers of oxidation in equine RBCs of L, M and H fractions. **(A)** Autofluorescence in green channel and **(B)** fluorescence intensity for monobromobimane (MBBR) and in L, M and H fractions of RBCs of 13 horses. Wilcoxon signed rank test was used for statistical analysis and the two-tailed “*p*” are presented as numbers.

RBCs harvested from the L and often H fraction of one horse was not enough to use any macroscopic method of detection of reduced glutathione. So, florescence readouts (autofluorescence and monobromobimane staining) were used to assess oxidized hemoglobin products and intracellular free thiols in RBCs using flow cytometry. As follows from Figure 6A, equine RBCs forming the H fraction showed higher auto-fluorescence in green and red channels compared to the cells of L and M fractions. Interestingly, the highest levels of reduced thiols were observed in RBCs from the M fraction, whereas cells from the L and H fractioned were more “oxidized” (Figure 6B).

### Band 4.1a:b Ratio

**Figure 7:**
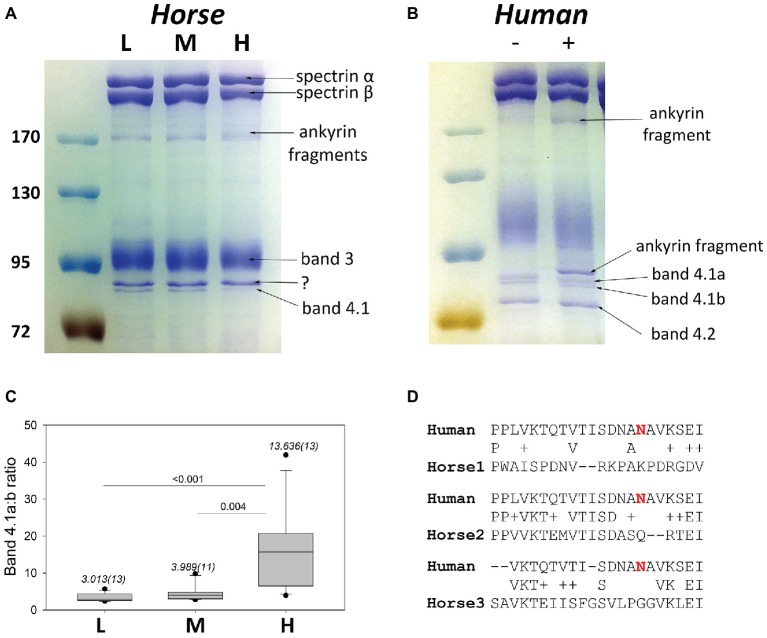
Band 4.1Ra:b potential as an aging marker in horses. **(A)** A representative SDS PAGE gel with equine membrane proteins isolated from L, M and H fractions. **(B)** A representative gel with human membrane proteins without (“−”) and with (“+”) proteolytic ankyrin cleavage. **(C)** Quantitative densitometry for the band 4.1a:b ratio for 11–13 horses (numbers in brackets). Shown are medians (numbers above the bars) and variance as well and the outcome of Wilcoxon signed rank test. **(D)** Sequence alignments for human and equine 4.1 protein sequences.

Equine membrane protein separation was performed using SDS PAGE. On the gels obtained for horse membrane proteins characteristic bands for spectrins, band 3 and band 4.1R-sized protein was obtained (Figure 7A). Densitometric ratio obtained for the “band 4.1a:b” was increasing with an increase in RBC density (Figure 7C) in equine RBCs. Apart of that, a faint band of about 180 kDa seen was identified as a product of ankyrin cleavage using mass spectrometry. This cleavage product could be detected in comparable amounts all fractions, L, M, and H. The presence of such band on the gels where human RBC membrane proteins were isolated (Figure 7B) revealed certain degree of proteolysis. If that happened (compare the left and right lanes in Figure 7B), an additional band, identified as ankyrin fragment was present above the band 4.1R double-band. We therefore performed mass spectrometry of the band 4.1-like duplet isolated from equine membranes and indeed found the presence of ankyrin fragment along with the band 4.1 protein. Sequence alignment of horse vs. human band 4.1 protein revealed the absence of the Asp in position corresponding to the Asn502 for all three isoforms of *EPB41* gene-related products of horse genome (Figure 7D). Deamidation of this Asn residue within the band 4.1R protein gives rise to the shift in electrophoretic mobility. It occurs progressively over the RBC life-span and is used as a biological clock showing age of RBCs of humans and other species (Inaba and Maede, 1988, 1992; Inaba et al., 1992). Isoforms 1 and 3 sequences for horses differ significantly from the human sequence in the vicinity of Asn502 having no Asn residues within it. Equine *EBP41* isoform 2 showing greater homology to the human *EBP41* has Asn502 replaced by Gln.

## Discussion

Equine RBC forming L, M, and H fractions are presented with several potentially age-related features that confirm the increase of RBC density with aging and may be used for identification of RBC longevity. These features include the differences in RBC size and morphology, membrane loss, changes in redox state and sub-cellular distribution, and of bulk levels of fluo-4 fluorescence intensity. Majority of methods used for detection of these parameters in our study require smaller volumes of blood and standard equipment that is often available in a specialized clinical laboratory such as a flow cytometer and a fluorescent microscope.

Aging of human RBCs is associated with a gradual loss of membrane, oxidation, and proteolytic cleavage of hemoglobin and cytoskeletal proteins (Lutz and Bogdanova, 2013). Younger human cells also have higher Ca^2+^ levels as recorded by fluo-4 fluorescence intensity (Figures 5C,E; Makhro et al., 2013). Persisting stress erythropoiesis gives rise to more immature and thus less stable RBC phenotype combining the features of young and senescent cells that have shorter life-span than the cells produced under conditions of basal erythropoiesis (Bogdanova et al., 2007). At the same time, in most studies in which erythropoiesis markers were studied in horses, stress erythropoiesis was induced by administration of pro-hemolytic compound phenylhydrazine, by phlebotomy or by recombinant human erythropoietin to increase the number of reticulocytes (Lumsden et al., 1975a,b; Radin et al., 1986; Cooper et al., 2005; Singh et al., 2007; McKeever et al., 2016). In these studies, focused on detection of (stress?) reticulocytes, a transient increase in both MCV and heterogeneity in RBC volume (RDW and MCV SD) was reported after induction of stress erythropoiesis. The only one study that, similar to us, used Percoll density gradient to isolate fractions of cells of low, medium, and high density, were assumed to be enriched with young, mature, and presumably senescent cells, reported a density-dependent change in the intracellular creatine content of RBCs (Wu et al., 1983). We have chosen similar approach when searching for further markers of RBC aging without triggering stress erythropoiesis based on characterization of equine RBCs forming L, M, and H fractions.

How do we know that the densest RBCs are also the oldest? The only ultimate marker accepted for human RBCs as a “biological clock” is the degree of deamidation of the band 4.1 protein. Deamidation of band 4.1 protein was also reported to occur in RBC membranes of several other mammalian species, including horses (Inaba and Maede, 1988, 1992; Inaba et al., 1992; Lutz et al., 1992). In order to prove the existence of deamidation, the authors used an antibody with a broad spectrum of inter-species cross-reactivity (Inaba and Maede, 1988) However, these findings raised some caution due to the lack of homology between the horse and human *EPB41* sequence (Figure 7C). The question, that remains unsolved, is on the nature of the shift in electrophoretic mobility for what may be two forms of EPB41 proteins in the absence of the main deamidation site (Asn502) in horses (Inaba et al., 1992; Inaba and Maede, 1992). Non-enzymatic Gln deamidation is occurring as well, but deamidation rate is approximately 100 times slower than that for Asn residues (Robinson and Robinson, 2004). Thus, it is unlikely that Gln deamidation in the homologous position within the equine band 4.1 protein would be a reliable marker of RBC age. Mass spectrometry revealed the presence of band 4.1 protein in these bands as well as ankyrin fragments, which may contribute to the alterations in density of the upper and lower band within 80 kDa range revealing an increase in cleavage of alpha-spectrin, band 3 protein, or ankyrin rather than modification of the band 4.1protein itself. Until the nature of changes leading to an increase in “band 4.1a:b ratio” along with cell density will be solved, we cannot use it as a reliable marker of equine RBC aging.

Characterization of the other “hallmarks of RBC aging” was then performed for the RBC forming L, M, and H fractions. The presence of cells positive for RNA was a reliable clinically relevant marker showing enrichment of the human L fraction with reticulocytes (Figure 1C), in line with the previous reports (Cooper et al., 2005). Earlier on the traces of one more marker of reticulocytes, CD71 (transferrin receptor), were reported to be associated not with RBCs, but with vesicles in serum of horses recovering from anemia caused by phlebotomy (Rout et al., 2015). We could not confirm or disapprove of the presence of any of reticulocyte markers in vesicles originating from these cells, as fractionation on Percoll density gradient is incompatible with this approach. Single vesicles, some of which contained larger Ca^2+^-filled compartments, similar to those seen in the H fraction, could be observed when whole blood was used for loading with fluo-4 and for life imaging (Figure 4B).

We could furthermore confirm that an increase in RBC density was at least in part caused by membrane loss. RBCs forming the H fractions were smaller in projected are (Figure 2D), deprived of band 3 protein and showed blunted response to hypoosmotic challenge (Figures 3E,G). Loss of CD71, ion pumps and channels and some other proteins accompany RBC membrane maturation, whereas loss of band 3 protein is a sign of aging and senescence of human RBCs (Lutz et al., 1988; Pantaleo et al., 2009; Lutz, 2012). We may therefore suggest that band 3 deprivation of equine RBCs forming the H fraction is a sign of senescence.

Oxidation was earlier on reported as a marker of RBC senescence [for review, see (Lutz and Bogdanova, 2013; Rifkind and Nagababu, 2013)]. Clusterization of oxidized band 3 protein is a marker tagging senescent RBCs for recognition by naturally occurring antibodies and clearance (Lutz, 2012). In horse blood, the densest RBCs showed signs of oxidative stress and inability to resist irreversible hemoglobin oxidation and damage (Figure 6A). Whereas the reduced thiol levels are comparable in cells from the L and H fraction (Figure 6B), increase in autofluorescence, known as a marker of hemoglobin oxidation and production of hemichromes (Kannan et al., 1988; Stoya et al., 2002) was consistent with age-related oxidation.

Finally, we have found alterations in basal intracellular free Ca^2+^ levels as well as the changes in its compartmentalization with an increase in density of equine RBCs. Aging of human RBCs is associated with the loss of activity and abundance of both Ca^2+^ pumps and Ca^2+^-permeable channels such as NMDA receptors in human RBCs (Luthra and Kim, 1980; Bogdanova et al., 2013). As a result free Ca^2+^ levels are reduced in dense senescent cells (Figures 4, 5; Bogdanova et al., 2013). For the equine RBCs as well increase in density was associated with a decrease in free Ca^2+^ levels (Figures 5D,F). Significant number of cells within the L fraction contained high Ca^2+^ cells with the nanovesicles filled with Ca^2+^, whereas the cells within the H fraction were presented with a single micrometer-sized compartment (Figures 4B,C). Earlier on production of two types of vesicles, microvesicles of ~150 nm and nanovesicles, ~50–60 nm in diameter was described in human RBCs in response to Ca^2+^ uptake (Salzer et al., 2002). Microvesicles contain low amounts of hemoglobin, Band 3 protein and glycophorins, and are enriched with acetylcholinesterase and stomatin. Nanovesicles are preferentially formed by lipid rafts and are hence enriched with stomatin as well as with proteins that are recruited to the rafts in response to Ca^2+^ uptake, synexin and sorcin (Salzer et al., 2002, 2008; Hagerstrand et al., 2006). Interestingly, mature equine RBCs are deprived of sphingomyelin (SM), compared to human, ovine, bovine, or porcine cells, although cholesterol levels are comparable (Wessels and Veerkamp, 1973). It is tempting to suggest, that SM could be lost from the membrane due to the effective maturation of equine RBCs. However, comparable or even lower SM levels have been shown for canine RBC membranes (Wessels and Veerkamp, 1973; Plasenzotti et al., 2007; Spengler et al., 2008), for which reticulocytes are easily detectible in blood (Bauer et al., 2012).

Precise control over the intracellular free Ca^2+^ and adaptations that prevent excessive Ca^2+^ accumulation in RBCs contribute to the prolongation of life expectancy of RBCs in human athletes (Bogdanova et al., 2013; Makhro et al., 2016). Majority of horses are physically active and seem to successfully cope with excess of intracellular Ca^2+^ in dense (and most likely senescent) RBCs by packaging it into the intracellular compartment. Thereby, activation of calpain and further unwanted effects related to Ca^2+^ overload are most likely prevented giving rise to an increase in life span for equine RBCs above that for humans (Figure 5, Table 1; Bogdanova et al., 2013).

## Limitations, Conclusions, and the Outlook

In our study we have performed detailed analysis of properties of equine RBCs as a function of the RBC density. Animals from which blood was collected were attending the Animal Hospital Zurich with various health issues not related to hematological phenotype. We furthermore did not discriminate between the warm and the coldblooded breeds, the age and gender of the animals. All these factors contributed to a larger variance, but revealed the robustness of the RBC aging markers, as they were shared by all the study participants. These markers included membrane loss, oxidation, and alterations in the intracellular free Ca^2+^ levels and its distribution pattern are robust and may be used as predictors of RBC age in horses.

The processes that contribute to maturation of young RBC in horses most likely involve very effective control over membrane loss and over the intracellular Ca^2+^ maintenance. As a result, premature damage is avoided and the longevity of RBCs is supported. Oxidative stress, as well as the growing density and stiffness of senescent horse RBC most likely trigger their clearance. More work, particularly at the level of bone marrow, has to be done to unravel the mysterious cause for of the lack of reticulocytes in peripheral blood of horses.

## Data Availability

All datasets generated for this study are included in the manuscript and/or the supplementary files.

## Ethics Statement

Equine heparinized blood samples from 19 horses were obtained from Clinical Laboratory of the Vetsuisse Faculty, University of Zurich. The samples were collected by veterinary practitioners as a part of diagnostic workup and sent to the laboratory for routine diagnostic purposes. Leftovers of the samples were used, and no additional blood volume was collected for the current study. No ethical approval was necessary for this study in compliance with the Swiss regulations. Blood samples were processed for analysis less than 12 h after blood withdrawal. Human blood samples were collected within the study on neocytolysis (DFG-SNF, # 320030E_180227) from four health male participants. The study involving human subjects was approved by the Ethics committee of the Medical Department of the University of Heidelberg (S-066/2018). Blood was collected by the medical practitioner at the Medical Department.

## Author Contributions

AB, AM, RH-L, and BR planned the study. AB supervised the study. SK, ES, and JB performed experiments. SK, AM, and AB analyzed the data. All the authors discussed the findings. AB and AM were writing the manuscript. All authors discussed the text and agreed with it.

### Conflict of Interest Statement

The authors declare that the research was conducted in the absence of any commercial or financial relationships that could be construed as a potential conflict of interest.
